# Mechanistic Contributions of Biological Cofactors in Islet Amyloid Polypeptide Amyloidogenesis

**DOI:** 10.1155/2015/515307

**Published:** 2015-10-20

**Authors:** Phuong Trang Nguyen, Nagore Andraka, Carole Anne De Carufel, Steve Bourgault

**Affiliations:** ^1^Department of Chemistry, Pharmaqam, University of Quebec in Montreal, Montreal, QC, Canada H3C 3P8; ^2^Quebec Network for Research on Protein Function, Structure, and Engineering (PROTEO), Canada; ^3^Biophysics Unit (CSIC, UPV/EHU) and Department of Biochemistry and Molecular Biology, Faculty of Science and Technology, University of the Basque Country, 48080 Bilbao, Spain

## Abstract

Type II diabetes mellitus is associated with the deposition of fibrillar aggregates in pancreatic islets. The major protein component of islet amyloids is the glucomodulatory hormone islet amyloid polypeptide (IAPP). Islet amyloid fibrils are virtually always associated with several biomolecules, including apolipoprotein E, metals, glycosaminoglycans, and various lipids. IAPP amyloidogenesis has been originally perceived as a self-assembly homogeneous process in which the inherent aggregation propensity of the peptide and its local concentration constitute the major driving forces to fibrillization. However, over the last two decades, numerous studies have shown a prominent role of amyloid cofactors in IAPP fibrillogenesis associated with the etiology of type II diabetes. It is increasingly evident that the biochemical microenvironment in which IAPP amyloid formation occurs and the interactions of the polypeptide with various biomolecules not only modulate the rate and extent of aggregation, but could also remodel the amyloidogenesis process as well as the structure, toxicity, and stability of the resulting fibrils.

## 1. Introduction

Several diseases are associated with the extracellular deposition of protein aggregates, including the Alzheimer's disease, the transthyretin, and light chain amyloidoses as well as type II diabetes [1]. Accumulation of insoluble protein in the extracellular space results from the aberrant assembly of proteins into aggregates, usually with a quaternary structure rich in cross-*β*-sheets, known as amyloid [2]. The causative link between the observed pathophysiology and amyloid formation is now supported by numerous genetic, biochemical, and pharmacological studies [1, 3–5]. More than 30 human endogenous proteins have been identified as precursors of amyloid fibrils whose deposition is associated with tissue degeneration. Although these amyloidogenic precursors share no sequence and native state structure homologies, amyloids extracted from patients share several structural, chemical, and biological features, including an extensive cross *β*-sheet structure and the capacity to bind specific dyes, such as Congo Red and thioflavin T (ThT) [1]. Particularly, fibrils are virtually always associated with nonfibrillar biomolecules, including the serum amyloid P component [6], apolipoprotein E [7], collagen [8], metals [9], glycosaminoglycans (GAGs) [10], and various lipids [11]. These cofactors were initially regarded as accessory molecules and/or contaminants of the amyloids. However, over the last two decades, several studies have instead highlighted that these amyloid cofactors can promote and/or modulate the amyloidogenic process. In this view, amyloid formation might not be simply a consequence of a protein misfolding event but may be more a consequence of the interaction of the amyloidogenic protein precursor with extrinsic factors and/or its (bio)chemical microenvironment [9].

The deposition of amyloid fibrils in the islets of Langerhans of patients afflicted by type II diabetes was originally described at the beginning of last century [12]. Over the 20th century, it was confirmed that islet hyalinization, that is, tissue degeneration into a classy translucent material, was closely associated with diabetes mellitus, particularly in elderly individuals [13, 14]. It is only in 1987 that the major component of islet amyloids was identified as a 37-residue peptide, the islet amyloid polypeptide (IAPP) [15] or amylin [16]. As IAPP is coexpressed, copackaged, and cosecreted with insulin by the pancreatic *β*-cells [17], the overproduction of insulin often associated with type II diabetes will lead to an increased release of IAPP. This elevated local concentration of IAPP in the islets of Langerhans should, in theory, promote the formation of amyloid. Nonetheless, although IAPP is expressed in nondiabetic subjects at levels higher than those required to form amyloids* in vitro* [18], IAPP rarely deposits in the pancreas of normal individuals [19]. This suggests that IAPP concentration is not the critical factor contributing to its aggregation and proposes that other elements could play a determinant role in the amyloidogenic process and, accordingly, in the etiology of type II diabetes.

In this review, we will initially describe IAPP structure and normal physiological functions and briefly present its proposed mechanisms of aggregation. We will mainly focus on the roles of amyloid cofactors and/or the biological environment in amyloid formation. As the role of model membranes in IAPP fibrillogenesis has been previously discussed in outstanding reviews [20–23], the present paper will mainly put an emphasis on other factors, such as GAGs and metals. Finally, we will discuss the potential roles of amyloid cofactors in *β*-cells degeneration associated with IAPP aggregation and amyloid deposition.

## 2. Islet Amyloid Polypeptide

**Figure 1:**
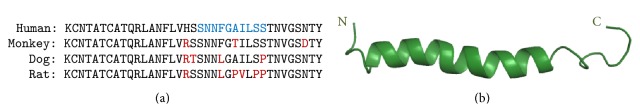
(a) Comparison of amino acid sequences of IAPP from different species. Residues that differ to those of human are indicated in red bold whereas the human 20–29 amyloidogenic segment is represented in bold blue. (b) Schematic ribbon representation of sodium dodecyl sulfate micelle-bound IAPP secondary structure (PDB code: 2KB8).

Characterization of the peptide isolated from human islet amyloids led to the identification of a C-*α*-amidated 37-residue peptide [15]. IAPP is expressed as an 89-residue polypeptide, called preproIAPP, containing a 22-residue signal peptide that is cleaved off in the reticulum endoplasmic to form proIAPP [24]. Subsequent posttranslational modifications of proIAPP involving the action of prohormone convertase (PC) enzymes and carboxypeptidase E (CPE) lead to the formation of a C-*α*-amidated, cyclized, and biologically active peptide [25]. The primary structure of IAPP has been determined in several mammalian species, including monkey, dog, mouse, and rat (Figure 1(a)). The N- and C-terminal regions of IAPP have been well conserved in all mammalian species, whereas the central 21–29 domain is more variable and shows important interspecies variations. Particularly, IAPP sequences found in mice and rat contain Pro residues at positions 25, 28, and 29 whereas the human sequence encompasses Ala, Ser, and Ser, respectively [26]. This variation is significant for the amyloidogenesis process, as rat (rIAPP) and mice (mIAPP) peptide are less prone to aggregation and these two species do not form islet amyloids [27]. In solution, human IAPP (hIAPP) exhibits a conformational ensemble mainly populated by disordered conformations, although it diverges from an absolute random coil by the presence of local and transient ordered structures [28]. For instance, the segment 5–19 of rIAPP, which exhibits a high homology with hIAPP, appears to transiently populate *α*-helix in its monomeric form [28, 29]. Besides, from molecular dynamics simulations, it was reported that hIAPP monomers could form ordered and extended *β*-hairpins [29]. In presence of lipid membrane models or organic solvent, such as hexafluoroisopropanol (HFIP), hIAPP readily adopts an *α*-helical conformation (Figure 1(b)) [30]. For example, in dodecylphosphocholine (DPC) micelles, rIAPP exhibits a structure characterized by a single helical region spanning from residues Ala-5 to Ser-23 followed by a disordered C-terminal domain [31]. When incubated with sodium dodecyl sulfate (SDS) micelles, hIAPP forms, instead, two *α*-helical segments spanning from residues Ala-7 to Val-17 and Asn-21 to Ser-28 and a short 3_10_ helix from Gly-33 to Asn-35 [32]. Both rat and human 1–19 IAPP fragments show a helical conformation in DPC micelles, although they adopt different orientation on the micelle surface [33].

IAPP is a member of the calcitonin peptide family, which includes calcitonin, calcitonin-gene-related peptides (CGRPs), and adrenomedullin [34]. IAPP shares 46% sequence homology with CGRP and 20% with calcitonin. These peptide hormones mediate their biological activities by binding and activating class B G protein-coupled receptors (GPCRs) [35]. Interestingly, no specific GPCR* per se* for IAPP has been identified so far. Instead, IAPP shares the calcitonin receptor (CT) with calcitonin, although it binds to CT with a relatively low affinity. The function, pharmacology, and selectivity of the CT receptor are altered by its association with receptor activity-modifying proteins (RAMPs). RAMPs constitute a family of single transmembrane proteins with 3 members: RAMP_1_, RAMP_2_, and RAMP_3_ [36]. Association of the CT receptor with RAMP_1_ or RAMP_3_ changes the selectivity of the receptor and increases significantly the affinity for IAPP [37]. IAPP specific binding sites were initially identified in the brain and the renal cortex and have now been identified in several peripheral tissues [38]. Under normal physiological conditions, IAPP is cosecreted with insulin from *β*-pancreatic cells in response to an elevated blood glucose concentration. In skeletal muscles, IAPP inhibits basal and insulin-stimulated glycogen synthesis, resulting in an increase of glucose-6-phosphate level [25]. Studies have also shown that IAPP suppresses glucagon secretion, decreases gastric emptying, and induces satiety [25, 39, 40]. IAPP may also be involved in the process of tissues calcification and could play a critical role in the inhibition of bone resorption [41]. Like other members of the calcitonin family, IAPP is a potent vasodilator and causes systemic hypotension and tachycardia [25, 42]. However, these effects were observed at much higher concentrations than the circulating physiological concentration of IAPP, normally ranging in the low picomolar (3–20 pM) [23, 43]. Thus, these effects should be interpreted with precaution since they could result from the activation of the CT receptor not associated with a RAMP and/or of the CT-receptor-like receptor. Taken together, the biological functions of IAPP are still far from being clearly understood [25].

## 3. IAPP Amyloid: Structure and Mechanisms of Formation

### 3.1. Structure of IAPP Amyloid Fibrils

**Figure 2:**
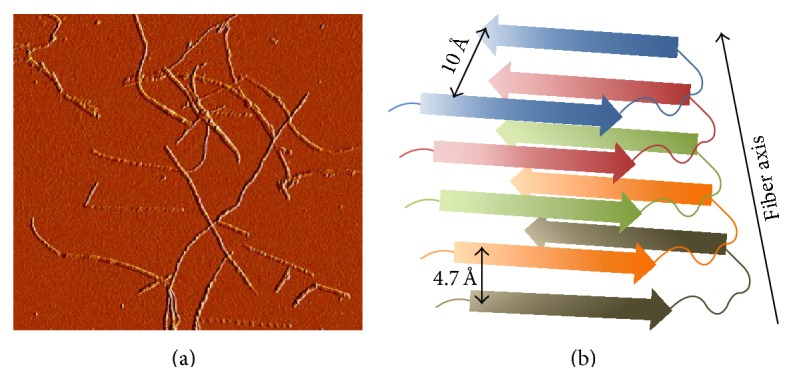
(a) Representative tapping mode atomic force microscopy image (amplitude mode) of IAPP amyloid fibrils prepared in homogenous solution (50 *μ*M at 37°C for 24 h.). (b) Schematic representation of the general cross-*β*-sheet quaternary structure of amyloids showing the interstrand (*≅*4.7 Å) and intersheet (*≅*10 Å) distances.

Amyloid fibrils, including IAPP amyloids, are highly ordered assemblies that predominantly adopt a characteristic cross-*β*-sheet quaternary structure [44]. This structural motif provides the most favorable organization for these supramolecular assemblies and can accommodate a high diversity of polypeptide sequences [45]. Amyloids are characterized by an X-ray diffraction pattern with two characteristic signals, a clear reflection at 4.7 Å along the direction of the fibril, and a diffuse reflection around 10 Å perpendicular to the fibril axis (Figure 2). By atomic force microscopy (AFM) and electron microscopy (EM), amyloids extracted from patients or prepared* in vitro* appear as long (0.5 to 10 *μ*m) and unbranched filaments having 4 to 15 nm of diameter [1] (Figure 2). Until recently, the structure of amyloids at the atomic level was unclear, since amyloids do not form crystals and are insoluble, precluding their characterization by X-ray crystallography and solution nuclear magnetic resonance (NMR). Thanks to recent advances in techniques such as solid state NMR [44] and the ability of growing nanocrystals of peptide fragments [46], it has been possible to elucidate the structure of several amyloids. These approaches, along with cryoelectron microscopy, have suggested that amyloid fibrils present a core sharing several characteristics. Nonetheless, it has also been reported that amyloids have significant structural differences [44]. These differences can be seen in (i) the length of the *β*-strands, (ii) the arrangement (parallel versus antiparallel) of the constituting sheets of the strand, (iii) the length and arrangement of structures which are not inside the fibril core, and (iv) the number of *β*-sheets per each protofilament [1]. Thus, although amyloid fibrils display similar characteristics, a marked polymorphism exists not only between fibrils from different precursors, but also between amyloids assembled from the same polypeptide but in different conditions [47].

The atomic structure of IAPP in its fibrillar form has been studied by a variety of approaches, including solid state NMR, X-ray crystallography, and electron paramagnetic resonance (EPR) spectroscopy. According to the technique used and/or the conditions in which IAPP amyloids were assembled, three main atomic models have been proposed. Firstly, in the model derived from solid state NMR study, IAPP protofibrils consist of two columns of symmetry related monomers packed against each other [48]. Each polypeptide monomer adopts a U-shaped structure and contains two *β*-strands connected by a bend-loop. These *β*-strands comprise, respectively, residues 8–17 and 28–37 whereas the loop involves residues 18–27 [48]. Residues 1 to 7 do not participate in the *β*-structure, most likely because of the conformational constraints imposed by the disulfide bridge [23]. Secondly, the Eisenberg group has proposed a model based on the crystallographic studies of IAPP fragments that shares many features with the solid state NMR model described above but differs in how the two columns of IAPP monomers pack against each other and in the length of the C-terminal *β*-strand [49]. Thirdly, EPR studies of IAPP variants lacking the Cys^2^–Cys^7^ disulfide bond have led to a slight variation of these two models. The protofibrils are still built up of U-shaped stacks of monomers, but the planes of the two *β*-strands within one IAPP molecule are staggered by around 15 Å [50]. Interestingly, in these three models, the 20–29 amyloidogenic segment is not part of a *β*-sheet. Instead, it forms a partially ordered bend that connects the two *β*-strands, questioning the sensitivity of hIAPP amyloid formation to substitutions and/or modifications within this amyloidogenic prone region [51]. Structural analysis of IAPP fibrils was so far exclusively performed using homogenous peptide assemblies, although amyloid deposits in islets of Langerhans of diabetic patients contain a variety of biomolecules, including GAGs, lipids, and other proteins. Thus, it will be interesting to study the molecular architecture of IAPP amyloids assembled in a biologically relevant heterogeneous environment.

### 3.2. Models of IAPP Amyloid Formation

While the mechanism by which proteins self-assemble into amyloids has been intensively studied over the last two decades, mechanistic details remain partially elusive and still the matter of controversy. Amyloidogenic polypeptides can be divided into two different structural classes: those that are intrinsically (or partially) disordered in their native state and those that show a well-defined tertiary structure in their monomeric soluble state. Generally, natively folded amyloidogenic proteins, such as transthyretin and *β*2-microglobulin, have to unfold (or misfold), at least partially, to form amyloids. In contrast, intrinsically disordered polypeptides, such as IAPP and A*β* peptide, need to undergo conformational rearrangements allowing the formation of locally ordered structure(s) to initiate the amyloidogenic process. The formation of amyloid fibrils is often described as a nucleation-dependent polymerization, although other models have been suggested [52], including the nucleated conformational conversion [53] and the monomer-directed conversion [54]. The nucleated polymerization model is characterized by the rate-limiting formation of the nucleus, which results from the equilibrium between monomers that are and are not assembly competent [52]. As soon as the nucleus is formed, assembly rapidly occurs by the addition of competent monomers to the growing end of the protofibrils. This model is characterized by two well-defined kinetics phases. Firstly, a low amount of dynamic and transient oligomeric species is produced in the lag phase. This phase takes place slowly because of the unfavorable interactions between monomers to form oligomers. Secondly, once the nucleus (competent oligomer) is formed, the elongation phase begins, leading to the rapid growth of the (bio)polymers [55]. Amyloid formation kinetics, seeding experiments as well as the difficulty of detecting low ordered oligomers [23], suggest that IAPP amyloidogenesis could be ascribed to a nucleated polymerization.

Recent studies performed with different amyloidogenic proteins have suggested that oligomers could be the most proteotoxic species of the aggregation cascade [56–58]. This hypothesis has prompted the biophysical investigation of the early steps in protein aggregation. For IAPP, two major models have been proposed for its oligomerization in homogenous solution: the helical intermediates model [59] and the *β*-hairpins model [60]. As inferred from NMR analysis and* in silico* prediction, monomeric soluble IAPP transiently adopts an *α*-helix between residues 5–19 [28] and it has been suggested that this helical intermediate could be on-pathway to amyloid formation. For instance, by analyzing the kinetics of *β*-sheet formation using two-dimensional infrared (2D IR) spectroscopy, it was observed that the disappearance of the random coil conformation was associated with the emergence of a *α*-helix [61]. Besides, the presence of a low percentage of HFIP, a solvent known to promote helical formation, in the aggregation solution of IAPP accelerates the rate of amyloid formation [62, 63]. Similarly, whereas the binding of IAPP to model membranes favors its initial conformational conversion from a random coil to a *α*-helix, it is well known that lipid vesicles significantly accelerate IAPP amyloid formation [64]. According to the helical intermediates hypothesis, self-association would be thermodynamically associated with helix formation within the 5–20 segment, in a similar way of the driven forces of coiled-coil motif formation [59]. In turn, this transient helical oligomer would generate a high local concentration of the C-terminal amyloidogenic segment of IAPP, favoring the intermolecular *β*-sheets formation. This *β*-sheet structure would then propagate leading to the formation of *β*-sheet rich supraassemblies [59]. Taking into account this model, several molecules have been recently designed to target and stabilize helical intermediates with the ultimate goal of inhibiting IAPP amyloid formation [65–68]. By combining ion mobility mass spectrometry and molecular dynamics simulations, it was instead proposed that IAPP early oligomerization steps include the formation of *β*-strand rich dimers [29, 60]. Bowers and colleagues have suggested that IAPP *β*-sheet-rich assemblies are formed from ordered beta-hairpins rather than from coiled structures. The discrepancy between these two models indicates that the initial events of IAPP amyloidogenesis still remain unclear. It is worth mentioning that in contrast to* in vitro* homogenous aqueous solution, the mechanisms of amyloid formation* in vivo* are most likely to be different and could involve alternative pathways. IAPP amyloidogenesis takes place in a heterogeneous and crowded environment with the potential interactions with several components of the extracellular matrix and the plasma membrane. Thus, mechanistic examinations of amyloid formation in heterogeneous environments constitute an important issue and relevant studies will now be discussed.

## 4. Biochemical Factors Modulating IAPP Amyloidogenesis

Amyloid formation has been originally perceived as a self-assembly homogeneous process in which the inherent physicochemical and structural properties of the amyloidogenic proteic precursor as well as its concentration constitute the major driving forces to fibrillation. Accordingly, the presence of biomolecules tightly associated with the amyloids* in vivo*, including GAGs, metals, glycoproteins, and lipids, was seen as a contamination of the fibrils occurring after aggregation and/or deposition. However, numerous biophysical investigations as well as* in vivo* biochemical studies have shown a prominent role of these extrinsic factors in amyloid deposition associated with the etiology of various diseases, including type II diabetes [1, 8, 9]. It is now evident that the biochemical microenvironment in which amyloid formation occurs and the interactions of the polypeptide precursor with various biomolecules not only modulate the rate and extent of aggregation, but also remodel the mechanisms as well as the structure, toxicity and stability of the resulting fibrils.

### 4.1. Glycosaminoglycans

Immunohistochemical analysis revealed that the basement membrane heparan sulfate proteoglycan (HSPG), perlecan, was present within islet amyloid deposits, suggesting a causative role of sulfated GAGs in IAPP fibrillogenesis [69]. Besides, incubation of hIAPP transgenic mouse isolated islets with WAS-406, an inhibitor of HSPGs synthesis, resulted in a dose-dependent decrease in amyloid formation [70]. Similarly, the Westermark group has established a mouse strain that overexpresses both hIAPP and heparanase, an enzyme that catalyzes the cleavage of cell surface heparan sulfate. They reported that isolated islets from these mice showed a marked reduction in amyloid accumulation upon a 2-week high glucose treatment; these conditions simulate the hyperglycemia observed in type II diabetes and stimulate IAPP expression and secretion [71]. In addition, since the original work by Castillo et al. [72], several reports have shown that sulfated GAGs, including heparin, heparan sulfate, and heparin derivatives, accelerate dramatically the rate of IAPP and pro-IAPP amyloid formation* in vitro* [73–77]. Overall, these studies constitute a clear testimony that sulfated GAGs could play an active role in islet amyloid deposition associated with type II diabetes.

**Figure 3:**
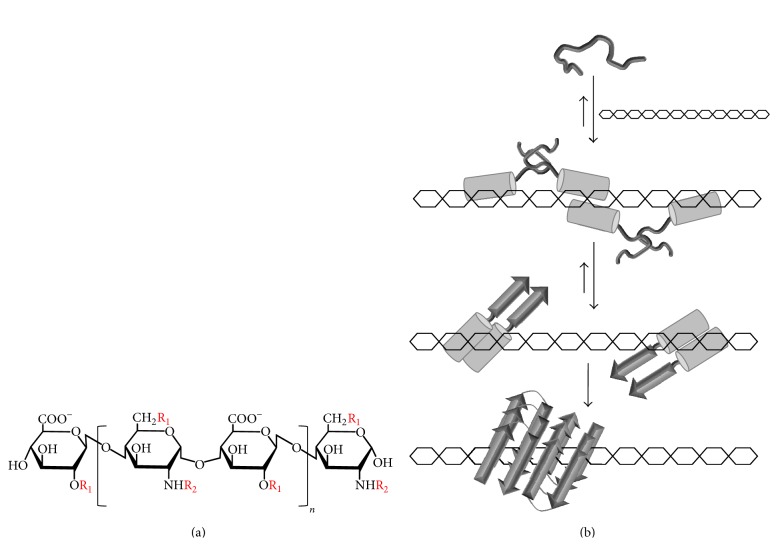
Roles of sulfated GAGs in IAPP amyloidogenesis. (a) Representative structure of heparin or heparan sulfate composed of glucuronic acid (GlcA) linked to glucosamine (GlcN) disaccharide repeating subunit. R_1_ could be –H or –SO_3_^−^ whereas R_2_ could be –H, –SO_3_^−^, or –COCH_3_. (b) Schematic representation of the postulated mechanism by which sulfated GAGs might promote IAPP amyloid formation. The positively charged N-terminal domain of IAPP binds to the sulfate moieties of GAGs by means of electrostatic interactions. This binding event triggers the formation of a *α*-helix (represented as a cylinder). This generates a high local concentration of peptide on the GAG scaffold that drives the association of IAPP amyloidogenic C-terminal segment, which has a high propensity to adopt a *β*-sheet (represented as an arrow). This drastically accelerates the formation of *β*-sheet rich assemblies.

GAGs are long and linear polysaccharides composed of repeating disaccharide units and some GAGs can contain up to 200 repeating disaccharide units [78]. They are abundant on the outer leaflet of the plasma membrane of every cell type of metazoan organisms and in their basement membrane and extracellular matrix (ECM) [79]. According to the structure of their carbohydrate backbone, GAGs can be classified into several classes. The most ubiquitous class of GAG is heparan sulfate (Figure 3(a)) which is expressed at the cell surface of nearly every cells, constituting more than 50% of total proteoglycans [80, 81]. Other types of GAGs include heparin, chondroitin sulfate, dermatan sulfate, keratan sulfate, and hyaluronic acid. Owing to their high density of carboxylate and sulfate groups, GAGs are highly negatively charged biopolymers that constitute a major reservoir of polyanions surrounding cells. With exception of hyaluronic acid and heparin, GAGs are usually covalently O-linked to a protein core, forming a structure known as proteoglycans. HSPGs, which constitute approximately 95% of all proteoglycans [82], are present in all tissues and comprise five types of protein core, including the cell surface syndecan and the ECM perlecan, the latter being a major constituent of pancreatic islet amyloids [69].

Over the last 15 years, several studies have demonstrated that the addition of sulfated GAGs to amyloidogenic proteins accelerates their fibrillogenesis* in vitro*. These polypeptides include both intrinsically disordered polypeptides such as the A*β* peptide [83], *α*-synuclein [84], and IAPP [72] and natively folded proteins such as transthyretin [85], gelsolin [86], and *β*2-microglobulin [87]. It has been proposed that GAGs hasten amyloidogenesis by a scaffold-based mechanism, in which the amyloidogenic protein, either in its monomeric or oligomeric form, interacts with the sulfated polysaccharides mainly through electrostatic interactions, increasing its local concentration and promoting aggregation [85, 86, 88]. It was also reported that the interaction with GAGs induces the conformational transition of the 3 kDa fragment of gelsolin [89] and A*β* peptide [83] from a random coil to *β*-sheet. However, this structural modification is most likely related to the aggregation process rather than to a conformational conversion within the monomeric protein.

The mechanisms by which sulfated GAGs accelerate IAPP amyloid formation have been studied by a combination of biophysical approaches and are similar to the one described for other amyloidogenic polypeptides. Owing to its net positive charge at physiological pH, IAPP can bind by means of electrostatic interactions with polyanionic sulfated GAGs. As a matter of fact, it was observed by NMR spectroscopy that heparin binds to the N-terminal segment of IAPP [73], which includes the only four potential positive charges in IAPP sequence: the *α*-amino group, Lys-1, Arg-11, and His-18. Besides, it was reported by isothermal titration calorimetry (ITC) that the affinity of IAPP to sulfated GAGs was dependent on the protonation state of His-18 and that the binding was predominately enthalpy-driven, most related to electrostatic interactions [75]. A heparin binding site was characterized within the N-terminal domain of proIAPP [77, 90] and it was suggested that the interaction of unprocessed proIAPP with sulfated GAGs could have strong implications for amyloid formation in pancreatic islets. FRET analyses between ThT and fluorescein-labelled heparin (FH) [73, 74] showed that IAPP binds to sulfated GAGs before amyloid formation, most likely in its monomeric form. As reported for other amyloidogenic polypeptides, heparin is incorporated into the fibrils and/or is tightly associated with the mature amyloids. By CD spectroscopy, IAPP and proIAPP association to sulfated GAGs induces a random coil to *α*-helix conformational conversion [75, 77] and this helical structure is rapidly converted into a *β*-sheet structure. As the binding of IAPP and proIAPP accelerates the rate of amyloid formation, this secondary conformational conversion supports the helical intermediates hypothesis described above. By using heparin analogs of different length and/or degree of sulfation, it was reported that the effects of GAGs on IAPP amyloidogenesis were dependent on the oligosaccharide length and sulfate content and not on the amount of charged monomers [72, 73]. Nonetheless, it was observed that the degree of sulfation of heparan sulfate isolated from pancreatic *β*-TC3 cells does not determine all aspects of GAG-mediated amyloid formation [91]. Besides, the nature of GAG backbone also affects, to some extent, the enhancement of IAPP fibril formation [72, 92]. Overall, these studies suggest a model for GAGs-accelerated IAPP amyloidogenesis in which the positively charged N-terminal segment of the peptide binds to the sulfate moieties of GAGs, inducing the formation of a *α*-helix. In turn, this generates a high local concentration of peptide on GAG scaffold that drives the association of IAPP amyloidogenic segment, accelerating drastically the formation of *β*-sheet rich assemblies (Figure 3(b)).

### 4.2. Metals

Several reports have suggested that the dysregulation of metal ion homeostasis could be implicated in the pathogenesis of amyloid diseases, comprising type II diabetes [9]. Binding sites for transition metals, including zinc, copper, and iron, have been characterized in numerous amyloidogenic polypeptides, such as A*β* peptide [93], *α*-synuclein [94], and *β*2-microglobulin [95]. Most of mechanistic studies have been so far performed with the A*β* peptides and have shown that physiological concentrations of metals, particularly Zn^2+^, are sufficient to accelerate the rate of amyloid formation, although divergent results were reported [96]. While it is known for more than 20 years that the secretory granules in pancreatic islets of Langerhans, which store IAPP and insulin, are characterized by a high concentration of zinc [97], the role of this metal in IAPP amyloidogenesis has not been addressed until recently [98, 99]. Particularly, it has been reported that zinc transport into *β*-cells secretory granules, involving the pancreas-restricted zinc transporter ZnT8, could play a significant role in the etiology of type II diabetes [100, 101]. This observation suggests that zinc homeostasis could be associated with IAPP misfolding/aggregation, although this avenue has not been explored* in vivo* so far.

The modulation of IAPP amyloidogenesis* in vitro* by zinc is complex and is dependent on zinc concentration as well as the pH and peptide concentration [98]. At pH 7.5, the presence of a low concentration of Zn^2+^ in the incubation solution decreases the rate of amyloid formation whereas at higher concentration the fibril elongation rate increases. It was also observed that while the total amount of fibrils is greatly reduced by zinc at all concentrations, the general morphology of the individual fibrils remained somewhat similar [98]. Notably, typical concentrations of zinc reported in the extracellular space where IAPP deposition occurs, ranging from 10 to 25 *μ*M [102], decrease significantly amyloid formation at physiological pH. In sharp contrast, at pH 5.5, at which the residue His-18 is protonated, zinc accelerated IAPP fibrillogenesis. Brender and colleagues have observed that IAPP in an organic solvent undergoes a structural conversion upon zinc binding characterized by a local disruption of the helical structure around residue His-18 [98]. Thus, the inhibitory effect of zinc observed at low concentrations was initially ascribed to the unfavorable incorporation of a charge inside the loops [98], as the imidazole ring of His-18 is located in the hydrophobic core of the fiber [48]. By combining ITC, NMR, and ESI mass spectrometry, it was observed that zinc favors the formation of off-pathway hexameric species while creating an energetic barrier for the formation of amyloids [103]. Thus, zinc binding to nonfibrillar IAPP with an affinity in the micromolar range [103] promotes the formation of prefibrillar aggregates [99], ultimately inhibiting the formation of amyloid fibrils.

The inhibition of amyloid formation by metals appears to be restricted to metals that are known as good ligands for histidine, such as Zn^2+^ and Cu^2+^, whereas Mg^2+^ and Ca^2+^, which are poor imidazole ligands, have no significant effect on IAPP amyloidogenesis [98, 104]. The effect of the buffer ion composition on the kinetics of IAPP amyloid formation was recently examined and it was reported that IAPP fibrillogenesis was dependent on the anion identity, while the nature of the cationic species has little effect on the rate of fibrils formation [105]. Overall, whereas the modulation of *α*-synuclein and A*β*-peptide amyloidogenesis by metals is well-documented, the role of metal homeostasis in islet IAPP deposition has been so far less studied and deserves further attention. Particularly, it will be interesting to probe the effects of zinc and copper on the kinetics of IAPP self-assembly in heterogeneous environment, that is, in presence of other biological factors such as GAGs and lipid membrane models.

### 4.3. Other Factors Modulating IAPP Amyloidogenesis

Virtually all amyloid deposits, including islet amyloids [106, 107], are associated with apolipoprotein E (apoE), a protein involved in lipid transport and metabolism. The importance of this protein in amyloid deposition has been highlighted in Alzheimer's disease as transgenic mice lacking the ApoE gene form only diffuse plaques but not mature neuritic plaques [108]. In sharp contrast, transgenic mice expressing hIAPP crossbred with apoE deficient mice showed similar prevalence and severity of islet amyloids, indicating that apoE is not a critical factor for islet amyloid deposition [109]. Nonetheless, it was observed* in vitro* that ApoE4 binds IAPP and inhibits amyloid formation [110]. Insulin, which is stored with IAPP in *β*-cell secretory granules, is one of the most potent inhibitors of IAPP aggregation [111]. Insulin binds to the putative helical domain of IAPP, stabilizing the compact isoform of IAPP and inhibiting the formation of *β*-sheets [112, 113]. The postulated mechanism of fibrillogenesis inhibition by insulin is consistent with the helical intermediates hypothesis. Anionic model membranes are the most studied biological cofactors in the context of IAPP amyloidogenesis, since they not only accelerate IAPP amyloid formation but they also recapitulate the postulated initial site of IAPP-induced cell death. The mechanisms of lipid-accelerated IAPP amyloidogenesis have been previously addressed in several excellent reviews [20–23, 114] and readers are invited to consult them for additional information.

## 5. Mechanism of IAPP Cytotoxicity

### 5.1. The Toxic Oligomeric Species Hypothesis

The presence of insoluble protein deposition in the pancreatic islets of patients suffering from type II diabetes has initially led to the postulate that amyloid fibrils cause *β*-cell degeneration [115]. This hypothesis was later reinforced by the work of Lorenzo and colleagues demonstrating the potential toxicity of IAPP fibrils on human pancreatic islet cells [116]. This cell death event was associated with membrane blebbing, chromatin condensation, and DNA fragmentation, indicating that IAPP amyloids trigger *β*-cell apoptosis. However, over the last fifteen years, several studies have instead suggested that nonfibrillar intermediates are the most toxic species of IAPP amyloid cascade. For instance, it was observed that the inhibition of amyloid fibril formation with rifampicin did not reduce IAPP-induced pancreatic cell death [117]. Furthermore, in a homozygous hIAPP transgenic mouse model, selective *β*-cell death and impaired insulin secretion were associated with the formation of early, small amorphous intra- and extracellular aggregates rather than with large amyloid deposits [118]. Bram and colleagues have recently reported the isolation of antibodies from diabetic patients that specifically recognized IAPP oligomers. Remarkably, these antibodies were shown to neutralize the apoptotic effect induced by IAPP cytotoxic species on *β*-cell [119]. Moreover, dynamic light scattering revealed that cytotoxicity corresponds to IAPP aggregates containing between 25 and 6 000 IAPP molecules [120]. Thus, as for other amyloid-related diseases, the scientific community generally agrees on the hypothesis that prefibrillar aggregates might be the toxic species causing *β*-cell death. However, considering that pancreatic islets from patients afflicted with type II diabetes are almost all converted into amyloids, this massive IAPP deposition most likely interferes with normal *β*-cell functions, such as insulin release [19]. Noteworthy, the search for the culprit species of the amyloidogenic cascade has been so far exclusively performed with aggregates prepared in IAPP homogenous solution. However, as described above, amyloid cofactors such as metals, GAGs, and lipids can remodel the pathway of aggregation and can lead to the formation of oligomer species with unusual morphological, physicochemical, and/or biological properties. Thus, it will be crucial in the nearest future to characterize the cytotoxicity of IAPP oligomers prepared in heterogeneous environment that reconstitutes, as possible, the extracellular environment of pancreatic islets.

### 5.2. Mechanisms of IAPP-Induced Cytotoxicity

Although the mechanisms by which IAPP induced *β*-cell death have been intensively investigated since IAPP discovery, the subject is very complex and is still the matter of debate. This topic has been recently discussed in excellent reviews [23, 121, 122] and, accordingly, we will briefly present the main postulated mechanisms. One of the most studied and accepted mechanisms is membrane disruption and transmembrane pore formation [20]. IAPP is a cationic peptide, favoring its electrostatic interaction with anionic lipids of the plasma membrane. Indeed, the nature of membrane model composition influences its aggregation [123]. Experiments performed with planar phospholipid bilayer membranes showed the formation of nonselective ion-permeable channels, suggesting that channel-like formation could trigger IAPP-induced cell death [124]. Similarly, the formation of abnormal vesicle-like membrane structures was observed when freshly dissolved IAPP was added to mouse and human islet cells [120]. Apoptosis, or programmed cell death, is another mechanism by which IAPP can cause *β*-cell death and is closely associated with membrane disruption. Actually, nonspecific channel-like formation by IAPP causes a high influx of Ca^2+^ inside the cell that can engage apoptosis [125, 126]. DNA fragmentation, a key apoptosis characteristic, was observable for RINm5F cells exposed to IAPP [127]. Moreover, IAPP induces p53 activation, a well-known tumor suppressing gene that regulates the cycle and increases the transcription of proapoptotic factors [128]. Similarly, it was observed that the gene encoding the G1 inhibitor p21 is upregulated when cells are incubated in presence of IAPP aggregates [127]. These studies suggest that IAPP can also trigger nonspecific apoptotic pathways. Besides, IAPP expression in islets upregulates the expression of the FAS receptor, a transmembrane protein able to engage programmed cell death, whereas the deletion of FAS reduced IAPP-induced toxicity [129], suggesting the involvement of specific apoptotic pathways.

Several studies have indicated that IAPP can induce pancreatic cell death by inducing the generation of reactive oxygen species (ROS). For instance, an increased level of ROS was observed when cells were exposed to IAPP oligomers [130]. Interestingly, it was observed that phycocyanin, a natural compound known for its antioxidant properties, protects pancreatic *β*-cells against IAPP-induced apoptosis by attenuating oxidative stress and modulating apoptotic pathways [130]. In contrast, treatment with the antioxidant N-acetyl-L-cysteine (NAC) prevented the rise of ROS induced by IAPP but did not prevent *β*-cell apoptosis [131]. Due to peripheral insulin resistance associated with type II diabetes, insulin and IAPP expression, maturation, and secretion by pancreatic *β*-cells are significantly increased [132]. This can overload the endoplasmic reticulum (ER), leading to ER stress and the activation of the unfolded protein response (UPR). For instance, an elevated expression of IAPP in hIAPP transgenic rats induces ER-stress, ultimately leading to *β*-cells apoptosis [133]. Interestingly, by establishing a mouse model overexpressing rIAPP at a comparable rate as the transgenic hIAPP mouse model, it was reported that the elevated ER stress depends on the propensity of IAPP to aggregate but is not the consequence of protein overexpression [134]. It was recently showed that the expression of hIAPP in mice with a *β* cell-specific autophagy defect results in an increase of *β*-cell dysfunction associated with IAPP-toxicity [135], suggesting a protective role of autophagy in type II diabetes. Overall, these studies indicate that IAPP-mediated cytotoxicity is multifaceted and is triggered by multiple mechanisms that are intrinsically related to each other.

### 5.3. Roles of Amyloid Cofactors in IAPP-Induced Cytotoxicity

Whereas biophysical studies have indicated that amyloid cofactors, including GAGs, metals, and lipids, can remodel IAPP aggregation landscape and biochemical investigations have suggested that oligomeric species induce *β*-cell death, it appears crucial to address the roles of these cofactors in IAPP-induced toxicity. It was observed that the coinjection of sulfated GAGs with IAPP in the culture media protects *β*-pancreatic cells against IAPP-mediated cytotoxicity [73, 75]. This result suggests that, by hastening amyloid formation, sulfated GAGs stimulate the formation of nontoxic fibrillar species, in agreement with the toxic oligomeric species hypothesis. The role of cell surface proteoglycans in IAPP-mediated cell death has been recently investigated. INS-1 cells treated with heparinase and chondroitinase in order to cleave polysaccharide chains of proteoglycans showed a similar vulnerability to IAPP to their nontreated counterpart [75]. This data indicates that the lack of GAGs on the outer leaflet of the plasma membrane does not prevent nor increases IAPP toxicity. This result was confirmed by means of the mutant CHO cell pgsA-745 [75], which lacks cell surface GAGs as a result of a deficiency in xylosyltransferase, a key enzyme in proteoglycans biosynthesis [136]. These observations are not in line with previous studies performed with the A*β* peptide showing that heparan sulfate deficient cells were essentially resistant to A*β* cytotoxicity [137]. Similarly, A*β* toxicity is attenuated in embryonic kidney cells overexpressing heparinase [137]. Nonetheless, as reported for IAPP, the removal of cell surface GAGs did not prevent HypF-N aggregates toxicity [138], suggesting some heterogeneity among the mechanisms of cell death induced by amyloidogenic polypeptides.

As described above, membrane disruption, including pore formation and membrane fragmentation, appears to play a key mechanistic role in the toxicity induced by IAPP on *β*-pancreatic cells. However, the contribution of the plasma membrane lipid composition and of its physicochemical properties on the cellular susceptibility towards IAPP has not been directly addressed so far. In an elegant work, Evangelisti and co-workers have recently shown that the extent of cytotoxicity of HypF-N oligomers is the result of a complex interplay between the physicochemical features of both the cell membrane and the oligomeric species [139]. Regarding IAPP, it was reported that depletion of cholesterol from plasma membrane of rat insulinoma cells inhibits the internalization of oligomers, which in turn potentiates IAPP cytotoxicity [140]. By means of real-time single particle tracking, it was shown that IAPP aggregates interact with GM1 gangliosides and decrease their lateral diffusion in neuroblastoma cell membrane [141]. As GM1 is a major constituent of membrane lipid rafts, which are known for their contribution to cell signaling pathways, it will be interesting to probe the role of GM1 in IAPP-induced toxicity. By combining biophysical approaches, it was shown that phosphatidylethanolamine (PE) phospholipids modulate the* in vitro* membrane disruption induced by IAPP [142]. This result suggests a possible role of PE in IAPP plasma membrane disruption, although this possibility has not been addressed* in vivo* so far. It was recently observed that copper interacts with IAPP to form metallopeptide complexes showing low toxicity towards pancreatic rat *β*-cells [143], indicating that metal ions can also modulate IAPP-induced cell death.

## 6. Conclusion

As summarized in this review, the role of the* so-called* accessory amyloid biomolecules in IAPP amyloidogenesis has been recently investigated by a combination of biophysical approaches. Regardless of the complexity of the microenvironment in which IAPP deposition occurs, the effects of several biological cofactors on amyloid formation are being increasingly recognized. Nonetheless, several issues should be addressed in order to better appreciate the implication of these biomolecules in the development of amyloid deposition. In turn, this knowledge should lead to deeper understanding of the mechanisms by which IAPP induced *β*-cell degeneration. Taking into account the prominent role of GAGs, metals, and lipids in IAPP amyloidogenesis, it will be particularly important that the identification of amyloid inhibitors* in vitro* is performed in milieu that recapitulates, as much as possible, the complex biological environment in which IAPP aggregation occurs. For instance, Hebda and colleagues have recently performed the screening of small molecules in presence of lipid membrane model and identified 36 molecules that were not previously reported as active toward IAPP fibril formation in homogenous solution [144]. Similarly, it was observed that the capacity of insulin to inhibit IAPP amyloidogenesis is significantly reduced in presence of sulfated GAGs [145] whereas the inhibition of IAPP fibrillogenesis by IS5, a small molecule alpha helix mimetic, is increased in presence of heparin [146]. Considering that the simplistic model of IAPP fibrillogenesis as a homogenous self-assembly process does not recapitulate amyloid deposition associated with the etiology of type II diabetes, it will be important in the future to develop* in vitro* experimental conditions to study IAPP aggregation that resemble the complexity of the pancreatic islet environment.

## References

[1] Chiti F., Dobson C. M. (2006). Protein misfolding, functional amyloid, and human disease. *Annual Review of Biochemistry*.

[2] Kelly J. W. (1998). The alternative conformations of amyloidogenic proteins and their multi-step assembly pathways. *Current Opinion in Structural Biology*.

[3] Sekijima Y., Wiseman R. L., Matteson J. (2005). The biological and chemical basis for tissue-selective amyloid disease. *Cell*.

[4] Coelho T., Maia L. F., Da Silva A. M. (2012). Tafamidis for transthyretin familial amyloid polyneuropathy: a randomized, controlled trial. *Neurology*.

[5] Cohen F. E., Kelly J. W. (2003). Therapeutic approaches to protein-misfolding diseases. *Nature*.

[6] Pepys M. B., Hirschfield G. M. (2003). C-reactive protein: a critical update. *The Journal of Clinical Investigation*.

[7] Kisilevsky R. (2000). The relation of proteoglycans, serum amyloid P and Apo E to amyloidosis current status, 2000. *Amyloid*.

[8] Bellotti V., Chiti F. (2008). Amyloidogenesis in its biological environment: challenging a fundamental issue in protein misfolding diseases. *Current Opinion in Structural Biology*.

[9] Alexandrescu A. T. (2005). Amyloid accomplices and enforcers. *Protein Science*.

[10] Ancsin J. B. (2003). Amyloidogenesis: historical and modern observations point to heparan sulfate proteoglycans as a major culprit. *Amyloid*.

[11] Gellermann G. P., Appel T. R., Tannert A. (2005). Raft lipids as common components of human extracellular amyloid fibrils. *Proceedings of the National Academy of Sciences of the United States of America*.

[12] Opie E. L. (1901). The relation Oe diabetes mellitus to lesions of the pancreas. Hyaline degeneration of the islands Oe langerhans. *The Journal of Experimental Medicine*.

[13] Bell E. T. (1959). Hyalinization of the islets of Langerhans in nondiabetic individuals. *The American Journal of Pathology*.

[14] Ehrlich J. C., Ratner I. M. (1961). Amyloidosis of the islets of Langerhans. A restudy of islet hyalin in diabetic and non-diabetic individuals. *The American Journal of Pathology*.

[15] Westermark P., Wernstedt C., Wilander E., Hayden D. W., O'Brien T. D., Johnson K. H. (1987). Amyloid fibrils in human insulinoma and islets of Langerhans of the diabetic cat are derived from a neuropeptide-like protein also present in normal islet cells. *Proceedings of the National Academy of Sciences of the United States of America*.

[16] Cooper G. J. S., Willis A. C., Clark A., Turner R. C., Sim R. B., Reid K. B. M. (1987). Purification and characterization of a peptide from amyloid-rich pancreases of type 2 diabetic patients. *Proceedings of the National Academy of Sciences of the United States of America*.

[17] Lukinius A., Wilander E., Westermark G. T., Engstrom U., Westermark P. (1989). Co-localization of islet amyloid polypeptide and insulin in the B cell secretory granules of the human pancreatic islets. *Diabetologia*.

[18] Jaikaran E. T. A. S., Nilsson M. R., Clark A. (2004). Pancreatic *β*-cell granule peptides form heteromolecular complexes which inhibit islet amyloid polypeptide fibril formation. *Biochemical Journal*.

[19] Westermark P. (2011). Amyloid in the islets of Langerhans: thoughts and some historical aspects. *Upsala Journal of Medical Sciences*.

[20] Brender J. R., Salamekh S., Ramamoorthy A. (2012). Membrane disruption and early events in the aggregation of the diabetes related peptide IAPP from a molecular perspective. *Accounts of Chemical Research*.

[21] Khemtémourian L., Killian J. A., Höppener J. W., Engel M. F. M. (2008). Recent insights in islet amyloid polypeptide-induced membrane disruption and its role in *β*-cell death in type 2 diabetes mellitus. *Experimental Diabetes Research*.

[22] Engel M. F. M. (2009). Membrane permeabilization by Islet Amyloid Polypeptide. *Chemistry and Physics of Lipids*.

[23] Cao P., Marek P., Noor H. (2013). Islet amyloid: from fundamental biophysics to mechanisms of cytotoxicity. *FEBS Letters*.

[24] Hutton J. C. (1994). Insulin secretory granule biogenesis and the proinsulin-processing endopeptidases. *Diabetologia*.

[25] Westermark P., Andersson A., Westermark G. T. (2011). Islet amyloid polypeptide, islet amyloid, and diabetes mellitus. *Physiological Reviews*.

[26] Betsholtz C., Christmansson L., Engstrom U. (1989). Sequence divergence in a specific region of islet amyloid polypeptide (IAPP) explains differences in islet amyloid formation between species. *FEBS Letters*.

[27] Westermark P., Engstrom U., Johnson K. H., Westermark G. T., Betsholtz C. (1990). Islet amyloid polypeptide: pinpointing amino acid residues linked to amyloid fibril formation. *Proceedings of the National Academy of Sciences of the United States of America*.

[28] Williamson J. A., Miranker A. D. (2007). Direct detection of transient alpha-helical states in islet amyloid polypeptide. *Protein Science*.

[29] Dupuis N. F., Wu C., Shea J.-E., Bowers M. T. (2009). Human islet amyloid polypeptide monomers form ordered *β*-hairpins: a possible direct amyloidogenic precursor. *Journal of the American Chemical Society*.

[30] Patil S. M., Xu S., Sheftic S. R., Alexandrescu A. T. (2009). Dynamic *α*-helix structure of micelle-bound human amylin. *The Journal of Biological Chemistry*.

[31] Nanga R. P. R., Brender J. R., Xu J., Hartman K., Subramanian V., Ramamoorthy A. (2009). Three-dimensional structure and orientation of rat islet amyloid polypeptide protein in a membrane environment by solution NMR spectroscopy. *Journal of the American Chemical Society*.

[32] Nanga R. P. R., Brender J. R., Vivekanandan S., Ramamoorthy A. (2011). Structure and membrane orientation of IAPP in its natively amidated form at physiological pH in a membrane environment. *Biochimica et Biophysica Acta—Biomembranes*.

[33] Nanga R. P. R., Brender J. R., Xu J., Veglia G., Ramamoorthy A. (2008). Structures of rat and human islet amyloid polypeptide IAPP_1−19_ in micelles by NMR spectroscopy. *Biochemistry*.

[34] Wimalawansa S. J. (1997). Amylin, calcitonin gene-related peptide, calcitonin, and adrenomedullin: a peptide superfamily. *Critical Reviews in Neurobiology*.

[35] Chapter M. C., White C. M., DeRidder A., Chadwick W., Martin B., Maudsley S. (2010). Chemical modification of class II G protein-coupled receptor ligands: frontiers in the development of peptide analogs as neuroendocrine pharmacological therapies. *Pharmacology & Therapeutics*.

[36] Hay D. L., Poyner D. R., Sexton P. M. (2006). GPCR modulation by RAMPs. *Pharmacology & Therapeutics*.

[37] Christopoulos G., Perry K. J., Morfis M. (1999). Multiple amylin receptors arise from receptor activity-modifying protein interaction with the calcitonin receptor gene product. *Molecular Pharmacology*.

[38] Paxinos G., Chai S. Y., Christopoulos G. (2004). In vitro autoradiographic localization of calcitonin and amylin binding sites in monkey brain. *Journal of Chemical Neuroanatomy*.

[39] Gedulin B. R., Rink T. J., Young A. A. (1997). Dose-response for glucagonostatic effect of amylin in rats. *Metabolism: Clinical and Experimental*.

[40] Scherbaum W. A. (1998). The role of amylin in the physiology of glycemic control. *Experimental and Clinical Endocrinology & Diabetes*.

[41] Naot D., Cornish J. (2008). The role of peptides and receptors of the calcitonin family in the regulation of bone metabolism. *Bone*.

[42] Zidverc-Trajkovic J., Stanimirovic D., Obrenovic R. (2009). Calcitonin gene-related peptide levels in saliva of patients with burning mouth syndrome. *Journal of Oral Pathology & Medicine*.

[43] Lutz T. A. (2010). The role of amylin in the control of energy homeostasis. *American Journal of Physiology—Regulatory Integrative and Comparative Physiology*.

[44] Tycko R., Wickner R. B. (2013). Molecular structures of amyloid and prion fibrils: consensus versus controversy. *Accounts of Chemical Research*.

[45] Nelson R., Sawaya M. R., Balbirnie M. (2005). Structure of the cross-*β* spine of amyloid-like fibrils. *Nature*.

[46] Makin O. S., Serpell L. C. (2005). Structures for amyloid fibrils. *The FEBS Journal*.

[47] Petkova A. T., Leapman R. D., Guo Z., Yau W.-M., Mattson M. P., Tycko R. (2005). Self-propagating, molecular-level polymorphism in Alzheimer's *β*-amyloid fibrils. *Science*.

[48] Luca S., Yau W.-M., Leapman R., Tycko R. (2007). Peptide conformation and supramolecular organization in amylin fibrils: constraints from solid-state NMR. *Biochemistry*.

[49] Wiltzius J. J. W., Sievers S. A., Sawaya M. R. (2008). Atomic structure of the cross-*β* spine of islet amyloid polypeptide (amylin). *Protein Science*.

[50] Bedrood S., Li Y., Isas J. M. (2012). Fibril structure of human islet amyloid polypeptide. *The Journal of Biological Chemistry*.

[51] Shim S.-H., Gupta R., Ling Y. L., Strasfeld D. B., Raleigh D. P., Zanni M. T. (2009). Two-dimensional IR spectroscopy and isotope labeling defines the pathway of amyloid formation with residue-specific resolution. *Proceedings of the National Academy of Sciences of the United States of America*.

[52] Kelly J. W. (2000). Mechanisms of amyloidogenesis. *Nature Structural Biology*.

[53] Lee J., Culyba E. K., Powers E. T., Kelly J. W. (2011). Amyloid-*β* forms fibrils by nucleated conformational conversion of oligomers. *Nature Chemical Biology*.

[54] Prusiner S. B. (1982). Novel proteinaceous infectious particles cause scrapie. *Science*.

[55] Harper J. D., Lansbury P. T. (1997). Models of amyloid seeding in Alzheimer's disease and scrapie: mechanistic truths and physiological consequences of the time-dependent solubility of amyloid proteins. *Annual Review of Biochemistry*.

[56] Bourgault S., Choi S., Buxbaum J. N., Kelly J. W., Price J. L., Reixach N. (2011). Mechanisms of transthyretin cardiomyocyte toxicity inhibition by resveratrol analogs. *Biochemical and Biophysical Research Communications*.

[57] Kayed R., Head E., Thompson J. L. (2003). Common structure of soluble amyloid oligomers implies common mechanism of pathogenesis. *Science*.

[58] Bemporad F., Chiti F. (2012). Protein misfolded oligomers: experimental approaches, mechanism of formation, and structure-toxicity relationships. *Chemistry & Biology*.

[59] Abedini A., Raleigh D. P. (2009). A critical assessment of the role of helical intermediates in amyloid formation by natively unfolded proteins and polypeptides. *Protein Engineering, Design & Selection*.

[60] Dupuis N. F., Wu C., Shea J.-E., Bowers M. T. (2011). The amyloid formation mechanism in human IAPP: dimers have beta-strand monomer-monomer interfaces. *Journal of the American Chemical Society*.

[61] Ling Y. L., Strasfeld D. B., Shim S.-H., Raleigh D. P., Zanni M. T. (2009). Two-dimensional infrared spectroscopy provides evidence of an intermediate in the membrane-catalyzed assembly of diabetic amyloid. *The Journal of Physical Chemistry B*.

[62] Padrick S. B., Miranker A. D. (2002). Islet amyloid: phase partitioning and secondary nucleation are central to the mechanism of fibrillogenesis. *Biochemistry*.

[63] Yanagi K., Ashizaki M., Yagi H., Sakurai K., Lee Y.-H., Goto Y. (2011). Hexafluoroisopropanol induces amyloid fibrils of islet amyloid polypeptide by enhancing both hydrophobic and electrostatic interactions. *The Journal of Biological Chemistry*.

[64] Knight J. D., Miranker A. D. (2004). Phospholipid catalysis of diabetic amyloid assembly. *Journal of Molecular Biology*.

[65] Saraogi I., Hebda J. A., Becerril J., Estroff L. A., Miranker A. D., Hamilton A. D. (2010). Synthetic *α*-helix mimetics as agonists and antagonists of islet amyloid polypeptide aggregation. *Angewandte Chemie—International Edition*.

[66] Kumar S., Brown M. A., Nath A., Miranker A. D. (2014). Folded small molecule manipulation of islet amyloid polypeptide. *Chemistry & Biology*.

[67] Kumar S., Miranker A. D. (2013). A foldamer approach to targeting membrane bound helical states of islet amyloid polypeptide. *Chemical Communications (Cambridge, England)*.

[68] Hassanpour A., de Carufel C. A., Bourgault S., Forgione P. (2014). Synthesis of 2,5-diaryl-substituted thiophenes as helical mimetics: towards the modulation of islet amyloid polypeptide (IAPP) amyloid fibril formation and cytotoxicity. *Chemistry*.

[69] Young I. D., Ailles L., Narindrasorasak S., Tan R., Kisilevsky R. (1992). Localization of the basement membrane heparan sulfate proteoglycan in islet amyloid deposits in type II diabetes mellitus. *Archives of Pathology & Laboratory Medicine*.

[70] Hull R. L., Zraika S., Udayasankar J. (2007). Inhibition of glycosaminoglycan synthesis and protein glycosylation with WAS-406 and azaserine result in reduced islet amyloid formation in vitro. *American Journal of Physiology—Cell Physiology*.

[71] Westermark G. T., Westermark P. (2011). Localized amyloids important in diseases outside the brain—lessons from the islets of Langerhans and the thoracic aorta. *The FEBS Journal*.

[72] Castillo G. M., Cummings J. A., Yang W. (1998). Sulfate content and specific glycosaminoglycan backbone of perlecan are critical for perlecan's enhancement of islet amyloid polypeptide (amylin) fibril formation. *Diabetes*.

[73] Jha S., Patil S. M., Gibson J., Nelson C. E., Alder N. N., Alexandrescu A. T. (2011). Mechanism of amylin fibrillization enhancement by heparin. *The Journal of Biological Chemistry*.

[74] Wang H., Cao P., Raleigh D. P. (2013). Amyloid formation in heterogeneous environments: islet amyloid polypeptide glycosaminoglycan interactions. *Journal of Molecular Biology*.

[75] de Carufel C. A., Nguyen P. T., Sahnouni S., Bourgault S. (2013). New insights into the roles of sulfated glycosaminoglycans in islet amyloid polypeptide amyloidogenesis and cytotoxicity. *Biopolymers*.

[76] Konno T., Oiki S., Morii T. (2007). Synergistic action of polyanionic and non-polar cofactors in fibrillation of human islet amyloid polypeptide. *FEBS Letters*.

[77] Abedini A., Tracz S. M., Cho J.-H., Raleigh D. P. (2006). Characterization of the heparin binding site in the N-terminus of human pro-islet amyloid polypeptide: implications for amyloid formation. *Biochemistry*.

[78] Bishop J. R., Schuksz M., Esko J. D. (2007). Heparan sulphate proteoglycans fine-tune mammalian physiology. *Nature*.

[79] Moremen K. W., Tiemeyer M., Nairn A. V. (2012). Vertebrate protein glycosylation: diversity, synthesis and function. *Nature Reviews Molecular Cell Biology*.

[80] Handel T. M., Johnson Z., Crown S. E., Lau E. K., Sweeney M., Proudfoot A. E. (2005). Regulation of protein function by glycosaminoglycans—as exemplified by chemokines. *Annual Review of Biochemistry*.

[81] Ihrcke N. S., Wrenshall L. E., Lindman B. J., Platt J. L. (1993). Role of heparan sulfate in immune system-blood vessel interactions. *Immunology Today*.

[82] Esko J. D., Selleck S. B. (2002). Order out of chaos: assembly of ligand binding sites in heparan sulfate. *Annual Review of Biochemistry*.

[83] McLaurin J., Franklin T., Zhang X., Deng J., Fraser P. E. (1999). Interactions of Alzheimer amyloid-*β* peptides with glycosaminoglycans: effects on fibril nucleation and growth. *European Journal of Biochemistry*.

[84] Cohlberg J. A., Li J., Uversky V. N., Fink A. L. (2002). Heparin and other glycosaminoglycans stimulate the formation of amyloid fibrils from *α*-synuclein in vitro. *Biochemistry*.

[85] Bourgault S., Solomon J. P., Reixach N., Kelly J. W. (2011). Sulfated glycosaminoglycans accelerate transthyretin amyloidogenesis by quaternary structural conversion. *Biochemistry*.

[86] Solomon J. P., Bourgault S., Powers E. T., Kelly J. W. (2011). Heparin binds 8 kDa gelsolin cross-*β*-sheet oligomers and accelerates amyloidogenesis by hastening fibril extension. *Biochemistry*.

[87] Relini A., de Stefano S., Torrassa S. (2008). Heparin strongly enhances the formation of *β*
_2_-microglobulin amyloid fibrils in the presence of type I collagen. *The Journal of Biological Chemistry*.

[88] Motamedi-Shad N., Monsellier E., Torrasa S., Relini A., Chiti F. (2009). Kinetic analysis of amyloid formation in the presence of heparan sulfate: faster unfolding and change of pathway. *The Journal of Biological Chemistry*.

[89] Suk J. Y., Zhang F., Balch W. E., Linhardt R. J., Kelly J. W. (2006). Heparin accelerates gelsolin amyloidogenesis. *Biochemistry*.

[90] Park K., Verchere C. B. (2001). Identification of a heparin binding domain in the N-terminal cleavage site of pro-islet amyloid polypeptide. Implications for islet amyloid formation. *The Journal of Biological Chemistry*.

[91] Hull R. L., Peters M. J., Perigo S. P., Chan C. K., Wight T. N., Kinsella M. G. (2012). Overall sulfation of heparan sulfate from pancreatic islet *β*-TC3 cells increases maximal fibril formation but does not determine binding to the amyloidogenic peptide islet amyloid polypeptide. *The Journal of Biological Chemistry*.

[92] Meng F., Abedini A., Song B., Raleigh D. P. (2007). Amyloid formation by pro-islet amyloid polypeptide processing intermediates: examination of the role of protein heparan sulfate interactions and implications for islet amyloid formation in type 2 diabetes. *Biochemistry*.

[93] Noy D., Solomonov I., Sinkevich O., Arad T., Kjaer K., Sagi I. (2008). Zinc-amyloid *β* interactions on a millisecond time-scale stabilize non-fibrillar Alzheimer-related species. *Journal of the American Chemical Society*.

[94] Yamin G., Glaser C. B., Uversky V. N., Fink A. L. (2003). Certain metals trigger fibrillation of methionine-oxidized *α*-synuclein. *The Journal of Biological Chemistry*.

[95] Calabrese M. F., Eakin C. M., Wang J. M., Miranker A. D. (2008). A regulatable switch mediates self-association in an immunoglobulin fold. *Nature Structural & Molecular Biology*.

[96] Hane F., Leonenko Z. (2014). Effect of metals on kinetic pathways of amyloid-beta aggregation. *Biomolecules*.

[97] Foster M. C., Leapman R. D., Li M. X., Atwater I. (1993). Elemental composition of secretory granules in pancreatic islets of Langerhans. *Biophysical Journal*.

[98] Brender J. R., Hartman K., Nanga R. P. R. (2010). Role of zinc in human islet amyloid polypeptide aggregation. *Journal of the American Chemical Society*.

[99] Brender J. R., Krishnamoorthy J., Messina G. M. L. (2013). Zinc stabilization of prefibrillar oligomers of human islet amyloid polypeptide. *Chemical Communications*.

[100] Rutter G. A., Chimienti F. (2015). SLC30A8 mutations in type 2 diabetes. *Diabetologia*.

[101] Sladek R., Rocheleau G., Rung J. (2007). A genome-wide association study identifies novel risk loci for type 2 diabetes. *Nature*.

[102] Aspinwall C. A., Brooks S. A., Kennedy R. T., Lakey J. R. T. (1997). Effects of intravesicular H^+^ and extracellular H^+^ and Zn^2+^ on insulin secretion in pancreatic beta cells. *The Journal of Biological Chemistry*.

[103] Salamekh S., Brender J. R., Hyung S.-J. (2011). A two-site mechanism for the inhibition of IAPP amyloidogenesis by zinc. *Journal of Molecular Biology*.

[104] Ward B., Walker K., Exley C. (2008). Copper(II) inhibits the formation of amylin amyloid in vitro. *Journal of Inorganic Biochemistry*.

[105] Marek P. J., Patsalo V., Green D. F., Raleigh D. P. (2012). Ionic strength effects on amyloid formation by amylin are a complicated interplay among debye screening, ion selectivity, and hofmeister effects. *Biochemistry*.

[106] Guan J., Zhao H.-L., Sui Y. (2013). Histopathological correlations of islet amyloidosis with apolipoprotein e polymorphisms in type 2 diabetic chinese patients. *Pancreas*.

[107] Charge S. B., Esiri M. M., Bethune C. A., Hansen B. C., Clark A. (1996). Apolipoprotein E is associated with islet amyloid and other amyloidoses: implications for Alzheimer's disease. *The Journal of Pathology*.

[108] Bales K. R., Dodart J. C., DeMattos R. B., Holtzman D. M., Paul S. M. (2002). Apolipoprotein E, amyloid, and Alzheimer disease. *Molecular Interventions*.

[109] Vidal J., Verchere C. B., Andrikopoulos S. (2003). The effect of apolipoprotein E deficiency on islet amyloid deposition in human islet amyloid polypeptide transgenic mice. *Diabetologia*.

[110] Lei P., Wu W.-H., Li R.-W. (2008). Prevention and promotion effects of apolipoprotein E4 on amylin aggregation. *Biochemical and Biophysical Research Communications*.

[111] Westermark P., Li Z.-C., Westermark G. T., Leckström A., Steiner D. F. (1996). Effects of beta cell granule components on human islet amyloid polypeptide fibril formation. *FEBS Letters*.

[112] Gilead S., Wolfenson H., Gazit E. (2006). Molecular mapping of the recognition interface between the islet amyloid polypeptide and insulin. *Angewandte Chemie—International Edition*.

[113] Susa A. C., Wu C., Bernstein S. L. (2014). Defining the molecular basis of amyloid inhibitors: human islet amyloid polypeptide-insulin interactions. *Journal of the American Chemical Society*.

[114] Engel M. F. M., Khemtémourian L., Kleijer C. C. (2008). Membrane damage by human islet amyloid polypeptide through fibril growth at the membrane. *Proceedings of the National Academy of Sciences of the United States of America*.

[115] Janciauskiene S., Ahrén B. (2000). Fibrillar islet amyloid polypeptide differentially affects oxidative mechanisms and lipoprotein uptake in correlation with cytotoxicity in two insulin-producing cell lines. *Biochemical and Biophysical Research Communications*.

[116] Lorenzo A., Razzaboni B., Weir G. C., Yankner B. A. (1994). Pancreatic islet cell toxicity of amylin associated with type-2 diabetes mellitus. *Nature*.

[117] Meier J. J., Kayed R., Lin C.-Y. (2006). Inhibition of human IAPP fibril formation does not prevent *β*-cell death: evidence for distinct actions of oligomers and fibrils of human IAPP. *American Journal of Physiology—Endocrinology and Metabolism*.

[118] Janson J., Soeller W. C., Roche P. C. (1996). Spontaneous diabetes mellitus in transgenic mice expressing human islet amyloid polypeptide. *Proceedings of the National Academy of Sciences of the United States of America*.

[119] Bram Y., Frydman-Marom A., Yanai I. (2014). Apoptosis induced by islet amyloid polypeptide soluble oligomers is neutralized by diabetes-associated specific antibodies. *Scientific Reports*.

[120] Janson J., Ashley R. H., Harrison D., McIntyre S., Butler P. C. (1999). The mechanism of islet amyloid polypeptide toxicity is membrane disruption by intermediate-sized toxic amyloid particles. *Diabetes*.

[121] Pillay K., Govender P. (2013). Amylin uncovered: a review on the polypeptide responsible for type II diabetes. *BioMed Research International*.

[122] Abedini A., Schmidt A. M. (2013). Mechanisms of islet amyloidosis toxicity in type 2 diabetes. *FEBS Letters*.

[123] Caillon L., Lequin O., Khemtémourian L. (2013). Evaluation of membrane models and their composition for islet amyloid polypeptide-membrane aggregation. *Biochimica et Biophysica Acta*.

[124] Mirzabekov T. A., Lin M.-C., Kagan B. L. (1996). Pore formation by the cytotoxic islet amyloid peptide amylin. *The Journal of Biological Chemistry*.

[125] Fawthrop D. J., Boobis A. R., Davies D. S. (1991). Mechanisms of cell death. *Archives of Toxicology*.

[126] Orrenius S., McCabe M. J., Nicotera P. (1992). Ca^2+^-dependent mechanisms of cytotoxicity and programmed cell death. *Toxicology Letters*.

[127] Zhang S., Liu J., Saafi E. L., Cooper G. J. S. (1999). Induction of apoptosis by human amylin in RINm5F islet *β*-cells is associated with enhanced expression of p53 and p21^WAF1/CIP1^. *FEBS Letters*.

[128] Mlynarczyk C., Fahraeus R. (2014). Endoplasmic reticulum stress sensitizes cells to DNA damage-induced apoptosis through p53-dependent suppression of p21(CDKN1A.). *Nature Communications*.

[129] Park Y. J., Lee S., Kieffer T. J. (2012). Deletion of Fas protects islet beta cells from cytotoxic effects of human islet amyloid polypeptide. *Diabetologia*.

[130] Li X.-L., Xu G., Chen T. (2009). Phycocyanin protects INS-1E pancreatic beta cells against human islet amyloid polypeptide-induced apoptosis through attenuating oxidative stress and modulating JNK and p38 mitogen-activated protein kinase pathways. *International Journal of Biochemistry and Cell Biology*.

[131] Zraika S., Hull R. L., Udayasankar J. (2009). Oxidative stress is induced by islet amyloid formation and time-dependently mediates amyloid-induced beta cell apoptosis. *Diabetologia*.

[132] Gedulin B., Cooper G. J. S., Young A. A. (1991). Amylin secretion from the perfused pancreas: dissociation from insulin and abnormal elevation in insulin-resistant diabetic rats. *Biochemical and Biophysical Research Communications*.

[133] Huang C.-J., Lin C.-Y., Haataja L. (2007). High expression rates of human islet amyloid polypeptide induce endoplasmic reticulum stress-mediated *β*-cell apoptosis, a characteristic of humans with type 2 but not type 1 diabetes. *Diabetes*.

[134] Huang C.-J., Haataja L., Gurlo T. (2007). Induction of endoplasmic reticulum stress-induced beta-cell apoptosis and accumulation of polyubiquitinated proteins by human islet amyloid polypeptide. *The American Journal of Physiology—Endocrinology and Metabolism*.

[135] Shigihara N., Fukunaka A., Hara A. (2014). Human IAPP-induced pancreatic *β* cell toxicity and its regulation by autophagy. *The Journal of Clinical Investigation*.

[136] Esko J. D., Stewart T. E., Taylor W. H. (1985). Animal cell mutants defective in glycosaminoglycan biosynthesis. *Proceedings of the National Academy of Sciences of the United States of America*.

[137] Sandwall E., O'Callaghan P., Zhang X., Lindahl U., Lannfelt L., Li J.-P. (2010). Heparan sulfate mediates amyloid-beta internalization and cytotoxicity. *Glycobiology*.

[138] Saridaki T., Zampagni M., Mannini B. (2012). Glycosaminoglycans (GAGs) suppress the toxicity of HypF-N prefibrillar aggregates. *Journal of Molecular Biology*.

[139] Evangelisti E., Cecchi C., Cascella R. (2012). Membrane lipid composition and its physicochemical properties define cell vulnerability to aberrant protein oligomers. *Journal of Cell Science*.

[140] Trikha S., Jeremic A. M. (2011). Clustering and internalization of toxic amylin oligomers in pancreatic cells require plasma membrane cholesterol. *The Journal of Biological Chemistry*.

[141] Calamai M., Pavone F. S. (2013). Partitioning and confinement of GM1 ganglioside induced by amyloid aggregates. *FEBS Letters*.

[142] Sciacca M. F. M., Brender J. R., Lee D.-K., Ramamoorthy A. (2012). Phosphatidylethanolamine enhances amyloid fiber-dependent membrane fragmentation. *Biochemistry*.

[143] Lee E. C., Ha E., Singh S. (2013). Copper(II)-human amylin complex protects pancreatic cells from amylin toxicity. *Physical Chemistry Chemical Physics*.

[144] Hebda J. A., Magzoub M., Miranker A. D. (2014). Small molecule screening in context: lipid-catalyzed amyloid formation. *Protein Science*.

[145] Wang H., Raleigh D. P. (2014). The ability of insulin to inhibit the formation of amyloid by pro-islet amyloid polypeptide processing intermediates is significantly reduced in the presence of sulfated glycosaminoglycans. *Biochemistry*.

[146] Hebda J. A., Saraogi I., Magzoub M., Hamilton A. D., Miranker A. D. (2009). A peptidomimetic approach to targeting pre-amyloidogenic states in type II diabetes. *Chemistry & Biology*.

